# Two types of lateral extension in juvenile nasopharyngeal angiofibroma: diagnostic and therapeutic management

**DOI:** 10.1007/s00405-014-2965-y

**Published:** 2014-03-06

**Authors:** Anna Szymańska, Marcin Szymański, Elżbieta Czekajska-Chehab, Małgorzata Szczerbo-Trojanowska

**Affiliations:** 1 Department of Interventional Radiology and Neuroradiology, Medical University of Lublin, Jaczewskiego 8, 20-954 Lublin, Poland; 2 Department of Otolaryngology Head and Neck Surgery, Medical University of Lublin, Jaczewskiego 8, 20-954 Lublin, Poland; 3 Department of Radiology, Medical University of Lublin, Jaczewskiego 8, 20-954 Lublin, Poland

**Keywords:** Computed tomography, Infratemporal fossa, Juvenile nasopharyngeal angiofibroma, Lateral extension, Magnetic resonance imaging, Pterygopalatine fossa

## Abstract

Juvenile nasopharyngeal angiofibroma is a benign, locally aggressive nasopharyngeal tumor. Apart from anterior lateral extension to the pterygopalatine fossa, it may spread laterally posterior to the pterygoid process, showing posterior lateral growth pattern, which is less common and more difficult to identify during surgery. We analyzed the routes of lateral spread, modalities useful in its diagnosis, the incidence of lateral extension and its influence on outcomes of surgical treatment. The records of 37 patients with laterally extending JNA treated at our institution between 1987 and 2011 were retrospectively evaluated. Computed tomography was performed in all patients and magnetic resonance imaging in 17 (46 %) patients. CT and MRI were evaluated to determine routes and extension of JNA lateral spread. Anterior lateral extension to the pterygopalatine fossa occurred in 36 (97 %) patients and further to the infratemporal fossa in 20 (54 %) patients. In 16 (43 %) cases posterior lateral spread was observed: posterior to the pterygoid process and/or between its plates. The recurrence rate was 29.7 % (11/37). The majority of residual lesions was located behind the pterygoid process (7/11). Recurrent disease occurred in 3/21 patients with anterior lateral extension, in 7/15 patients with both types of lateral extensions and in 1 patient with posterior lateral extension. JNA posterior lateral extension may spread behind the pterygoid process or between its plates. The recurrence rate in patients with anterior and/or posterior lateral extension is significantly higher than in patients with anterior lateral extension only. Both CT and MRI allow identification of the anterior and posterior lateral extensions.

## Background

Juvenile nasopharyngeal angiofibroma (JNA) is a vascular tumor that originates in the sphenopalatine foramen, at the area of the pterygoid canal aperture [1]. The disease is rare, accounting for 0.05 % of all head and neck tumors, and most often occurs in adolescent males [2–6]. JNA is a unique clinical problem due to the controversy regarding its exact nature, pathogenesis and best treatment option for most advanced tumors.

Although benign, JNA behaves aggressively, extending to the adjacent areas. From its site of origin the tumor grows medially to the nasopharynx, where it usually becomes symptomatic. Some tumors spread laterally to the pterygopalatine fossa and, with further growth, to the infratemporal fossa. JNAs with large lateral extension in the infratemporal fossa and cheek without medial spread to the nasopharynx are very rare [7].

Expanding tumor may also grow along the skull base posterior to the pterygoid process, which may lead to parapharyngeal space involvement. This type of lateral growth should be identified as a posterior lateral extension, in contrast to the anterior lateral extension in the pterygopalatine fossa, which is more common and easier to identify during surgery. Extranasopharyngeal extensions of JNA include also the nasal cavity, paranasal sinuses, the orbit and in advanced cases also the cranium.

Surgical removal is the gold standard treatment for extracranial angiofibromas [5, 6, 8]. Extranasopharyngeal extensions may increase the risks of surgical treatment due to potential complications of wide surgical exposure, intraoperative hemorrhage and recurrence. In this context, the problem of the posterior lateral extension is of particular interest.

The aim of this study was to analyze radiological findings of JNA patients with lateral extension with special attention given to routes of lateral spread and modalities useful in its diagnosis. The incidence of lateral spread and its influence on planning and outcomes of surgical treatment were also discussed.

## Materials and methods

A retrospective chart review of 47 patients with juvenile nasopharyngeal angiofibroma referred to our institution between 1987 and 2011 was performed. The records of 37 patients with tumors extending laterally from the nasopharynx were included in the study. The study was approved by the institutional ethical committee.

All patients in the selected group were males. The age ranged from 9 to 30 years (the median age was 16 years). Most common presenting symptoms were nasal obstruction in 34 of 37 patients (92 %) and epistaxis in 32 of 37 patients (86 %). Additional complaints were facial swelling in four patients and proptosis in two patients. The duration of symptoms ranged from 2 to 24 months.

Preoperative imaging workup included contrast-enhanced computed tomography (CT) in all cases and magnetic resonance imaging (MRI) in 17 (46 %) cases. In 20 patients MRI was not performed, because they were diagnosed before MRI was available at our institution or information provided by CT was sufficient for the surgeon. Twenty nine CT and 14 MRI studies were performed at our institution and the radiological equipment included 32-slice scanner system Lightspeed PRO32 and 64 slice CT scanner system (both GE Healthcare global imaging, Dewaukee, WI, USA) and two 1.5 T MRI systems: Edge Eclipse (Picker International Inc, Cleveland, Ohio, USA) and Magnetom Avanto (Siemens AG, Munchen, Germany). The remaining patients had imaging studies from referring institutions performed on different types of MR and CT scanners. Although some scanning parameters varied, all MR studies were performed on 1.5 T systems, with multiplanar imaging using unenhanced T2-weighted, T1-weighted and gadolinium-enhanced T1-weighted sequences. In all patients CT scans with contrast-enhanced axial sections were available.

Carotid angiography was performed in all cases and preoperative embolization in 27/37 (72.9 %) cases.

Two radiologists reviewed the CT and MR images to determine the routes and extension of JNA lateral spread and compare the usefulness of CT and MRI for depicting this growth direction. Images were analyzed for erosion and dislocation of bony structures, widening of the foramina of the cranial base, obliteration of normal fatty spaces and tumor contrast enhancement. All tumors were staged according to Andrews classification [9].

Six patients presented with recurrent tumors after operations in other hospitals. All 37 patients underwent surgical treatment at our institution and were operated on by two main surgeons. Statistical analysis of recurrence rates in patients with various types of lateral extensions was performed (*χ* ^2^ test with Yates correction).

## Results

Thirty-seven patients with laterally extending JNA were included in this study. According to Andrews classification, 14 (38 %) patients had stage II, 15 (40 %) had stage IIIa, 6 (16 %) had stage IIIb, 1 (3 %) had stage IVa and another (3 %) had stage IVb. Tumor radiological extensions in the presented group are displayed in Table 1. Intracranial spread occurred in 1 of 21 (4.7 %) patients with only anterior lateral extension (group A) and in 7 of 15 (53.3 %) patients with both types of lateral extensions (group B) (Table 2).

**Table 1:** Tumor radiological extensions in the 37 presented cases

Anterior lateral extension 36/37 (97 %)	Posterior lateral extension 16/37 (43 %)	Other
36/37 (97 %): pterygopalatine fossa	7/37 (19 %): between pterygoid plates	8 (22 %): intracranial
		34 (91.8 %): vidian canal
20/37 (54 %): infratemporal fossa	4/37 (11 %): posterior to the pterygoid process	15 (40 %): cancellous bone at the pterygoid base
	5/37 (13 %): both types	8 (22 %): orbit
		4 (11 %): cheek

**Table 2:** Summary of types of lateral extensions, intracranial spread and tumor recurrences in the presented cases

Total number of patients	Localization of the lateral extension		Intracranial spread no. (%)	Recurrences	
	No (%)	Type of lateral extension		No. (%)	Location
37	21/37 (56.8 %)	Anterior (group A)	1/21 (4.7 %)	3/21 (14.2 %)	3-Pterygoid base
	15/37 (40.5 %)	Anterior and posterior (group B)	8/15 (53.3 %)	7/15 (46.6 %)	6-Posterior to PP 1-Intracranial
	1/37 (2.7 %)	Posterior (group C)	–	1	1-Posterior to PP

*PP* pterygoid process

### Routes of lateral spread

**Fig. 1:**
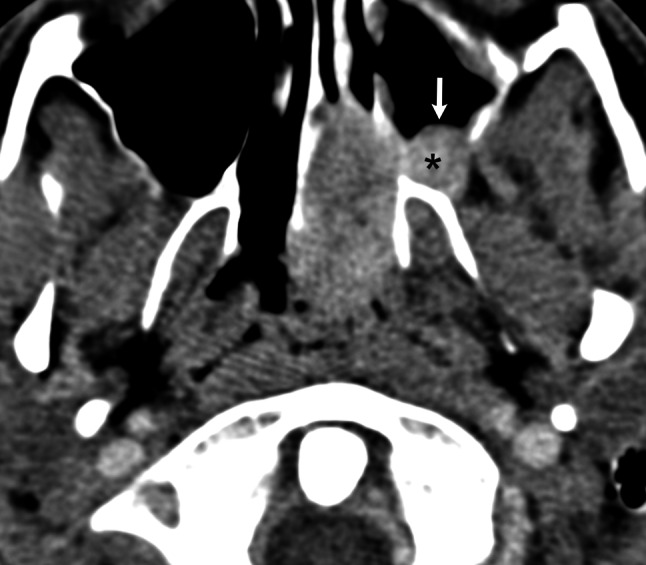
Contrast-enhanced axial CT scan indicates forward displacement of the posterior maxillary wall (*arrow*) and tumor invasion of the pterygopalatine fossa (*asterisk*)

In 36/37 (97 %) patients tumor spread laterally to the pterygopalatine fossa showing anterior lateral spread pattern (Fig. 1). Of these, in 20 (54 %) patients further growth into the infratemporal fossa was stated.

**Fig. 2:**
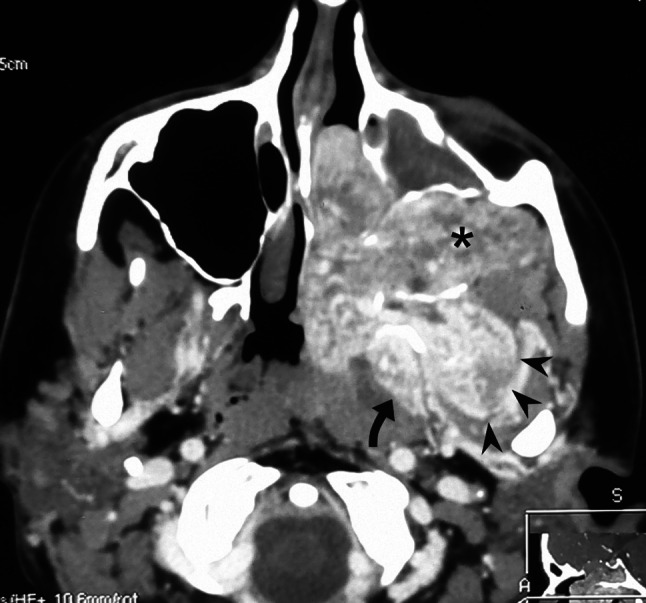
Contrast-enhanced axial CT scan well demonstrates three routes of tumor lateral spread in a case of extensive JNA: into the pterygopalatine and infratemporal fossa (*asterisk*), posterior to the pterygoid process (*curved arrow*) and between its plates (*arrowheads*)

In 16/37 (43 %) cases, posterior lateral spread with growth vector directed posteriorly to the pterygoid process was observed. In 7/37 (19 %) patients tumor eroded the pterygoid process and extended laterally between its plates. In 4/37 (11 %) patients, lateral extension posterior to the pterygoid process was found. In 5/37 (13 %) cases, both the above-mentioned ways of posterior lateral spread were stated (Table 1) (Fig. 2).

Of the whole group, in 21/37 (56.8 %) patients only anterior lateral spread was observed. Fifteen (40.5 %) patients had both anterior and posterior lateral extensions and one patient had only posterior lateral extension (Table 2).

### Radiological findings

**Fig. 3:**
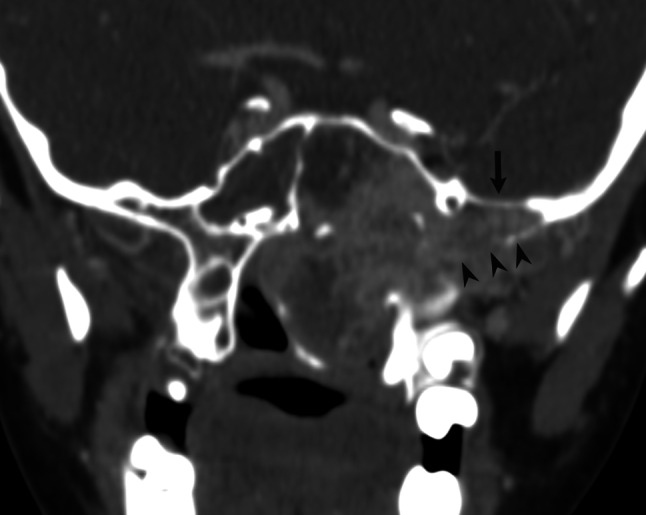
Coronal CT scan indicates extensive involvement and expansion of the pterygoid base and greater sphenoid wing diploë (*arrowheads*) with erosion of the skull base (*arrow*)

In 36/37 (97 %) patients, anterior bowing of the posterior maxillary wall and tumor involvement of the pterygopalatine fossa were observed. CT showed erosion of the pterygoid process and widening of the sphenopalatine foramen in 36/37 (97 %) cases. Advanced destruction of the pterygoid process was stated in 20/37 (54 %) patients. In 15/37 (40 %) patients, tumor invaded cancellous bone at the base of the pterygoid process or the greater wing of the sphenoid (Fig. 3). Involvement of the Vidian canal occurred in 34/37 (91.8 %) patients in the whole group, including 18/21 (85.7 %) with anterior lateral extension and all 16 (100 %) patients with anterior and/or posterior lateral extension.

All CT and MR images showed intensive tumor enhancement after contrast administration. Both modalities in all cases well depicted the presence of anterior lateral extension and in all cases allowed the identification of the posterior lateral spread. However, the relationship of posterior lateral extensions to the eroded and displaced pterygoid process was better demonstrated on CT scans than on MRI. Both methods allowed accurate delineation of tumor margins and the extension of the posterior lateral spread, as well as anterior lateral spread in the pterygopalatine and infratemporal fossa. Both CT and MRI allowed visualization and precise definition of the medullary bone involvement of the sphenoid.

### Surgical treatment and outcomes

Surgical treatment was applied in 37 cases. The following approaches were used: transpalatal in 6 cases, sublabial degloving in 20 cases and infratemporal fossa approach in 5 cases. In six patients endoscopic resection was performed. Follow-up ranged 1–25 years. In all patients the diagnosis of JNA was histopathologically confirmed.

**Fig. 4:**
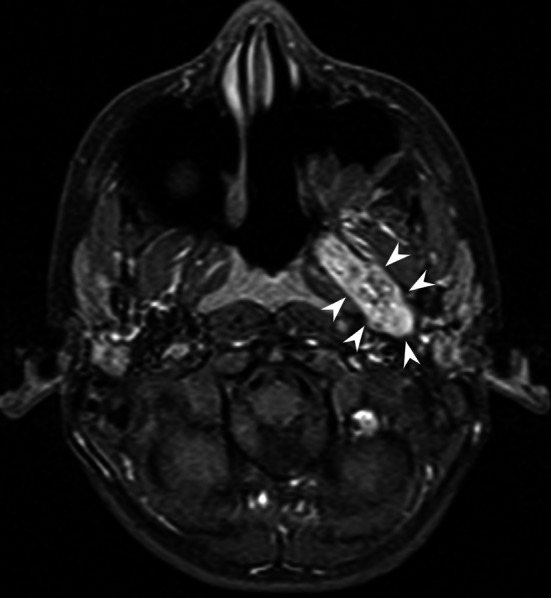
Axial contrast-enhanced T1-weighted MR image with fat saturation demonstrates residual tumor between the pterygoid plates (*arrowheads*)

The recurrence rate was 29.7 % (11/37). Residual tumor was located at the base of the pterygoid process in 3/11 (27.2 %) patients and posteriorly to the pterygoid process in 7/11 (63.6 %) patients (including three patients with persistent lesion located between the pterygoid plates) (Fig. 4). One patient (1/11–9.0 %) had a residual intracranial extension, which was removed in a second-stage neurosurgical operation. Except for one case of asymptomatic stable residual mass under 10 years follow-up, all residual tumors were successfully surgically removed.

Recurrent disease was observed in 3 out of 21 (14.2 %) patients with anterior lateral extension via the pterygopalatine fossa, in 7 out of 15 (46.6 %) patients with both types of lateral extensions and in 1 patient with exclusively posterior lateral extension (Table 2). The recurrence rate in patients with posterior lateral extension and both types of lateral spread (groups B and C) was significantly higher than in patients with anterior lateral extension only (group A) (*p* < 0.05).

Surgery included drilling of the vidian canal and basis sphenoid in 27/37 patients with tumor involvement of these structures. In the remaining 10/37 cases, available medical records did not contain detailed surgical information about drilling of this area; therefore its potential influence on the recurrence rate could not be assessed. However, the three cases of residual tumor at the pterygoid base occurred in the latter group.

## Discussion

JNAs show locally invasive nature and tend to extend into the natural foramina, fissures and sinus compartments.

From the area of the sphenopalatine foramen, the tumor grows laterally to the pterygopalatine fossa and with further progression to the infratemporal fossa, through the enlarged pterygomaxillary fissure. This extension results in a widening of the sphenopalatine foramen and anterior bowing of the posterior maxillary wall, with or without posterior displacement of the pterygoid process [4, 10, 11]. These features together with erosion of the pterygoid process and enhancing tumor mass in the nasopharynx and pterygopalatine fossa are characteristic signs of JNA. In our experience these radiological findings are well recognized on both CT and MRI. While CT provides precise definition of bony changes, MRI allows better evaluation of the intracranial spread, as well as superior differentiation between tumor mass and mucosal swelling or fluid retention in paranasal sinuses.

Lateral extension of the tumor to the pterygopalatine fossa is a common finding. It was reported by Rekonnen et al. [6] in 25.9 % of cases, Economou et al. [12] in 53 % of cases and Antonelli et al. [13] in 73 % of cases. In our study initial evaluation of 47 patients treated at our institution between 1987 and 2011 revealed the presence of tumor in the fossa pterygopalatine in 76.5 % of patients and further involvement of the infratemporal fossa in 54 % of patients.

Although tumor growth pattern with tendency to occupy the pterygopalatine fossa has been well described, few authors present cases of JNA with lateral spread posterior to the pterygoid process. This route of lateral growth was observed in 2 of 23 cases (9 %) reported by Radkowski et al. [14] and in 8 of 20 cases (40 %) reported by Hyun et al. [3]. In our study apart from lateral spread posterior to the pterygoid process, we identified route of spread between pterygoid plates, which was an apparent source of residual disease in three patients. Our primary retrospective evaluation of 47 patients with JNA treated at our institution revealed the presence of lateral extension posterior to the pterygoid process and/or between its plates in 16 (34 %) patients. In the analyzed group of 37 patients with lateral spread, almost half of patients (40.5 %) presented with both anterior and posterior lateral extensions. Our results show that posterior lateral extension usually follows anterior lateral spread, but rarely it may be observed without involvement of the pterygopalatine fossa.

In contrast to anterior lateral spread to the pterygopalatine fossa, posterior lateral extension occurs less frequently and is more difficult to identify due to variable patterns of tumor expansion and bony changes at the area of the pterygoid process. Therefore, some small lesions may be missed on imaging studies. The surgical implications of the posterior lateral spread demand a thorough evaluation of tumor extensions posterior and/or between pterygoid plates. In our experience, contrast-enhanced CT and MRI both allow identification of the posterior lateral extension. However, precise evaluation of the relationship between tumor extensions and displaced/eroded pterygoid process requires the best bone imaging available, provided by CT. This modality also provides the surgeon with precise visualization of the anatomical bony landmarks.

**Fig. 5:**
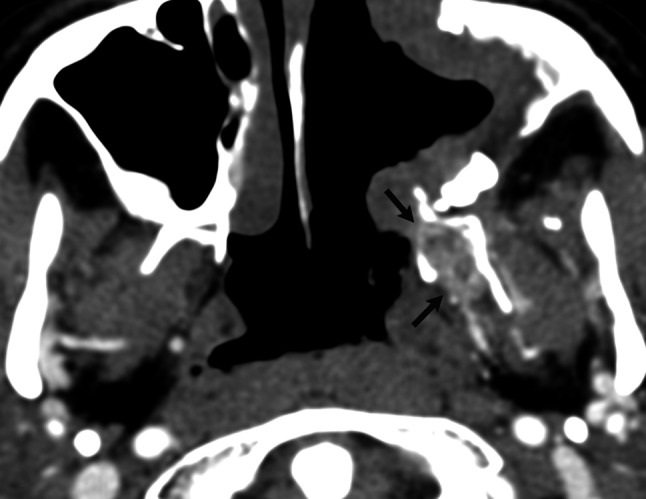
Axial CT scan after application of contrast medium shows small, submucosal residual tumor between the pterygoid plates (*arrows*)

It is generally accepted that surgical resection is the treatment modality of choice for JNA [15]. When choosing the surgical approach the following points should be considered: adequate exposure of tumor extensions, vascular control of the tumor and minimal invasiveness with respect to soft tissue and bony disruption. Various surgical methods have been used for complete and safe resection of JNAs. The four approaches used at our institution were transpalatal approach, midfacial degloving, the infratemporal fossa approach and endoscopic technique. *Transpalatal approach* is best suited for tumors limited to the nasopharynx, nasal cavity and sphenoid sinuses. It has an advantage of no facial incisions, but the main drawback is limited lateral exposure. At our institution it has been replaced by endoscopic removal. *Midfacial degloving* allows resection of tumors invading paranasal sinuses, orbit, pterygopalatine and infratemporal fossae, except for lesions with large infratemporal fossa extensions (up to temporal fossa) [8, 15]. Due to the use of sublabial and intranasal incisions, it allows good surgical exposure without facial scars. *The infratemporal fossa approach* provides access to most extensive tumors, involving the infratemporal fossa, middle cranial fossa and lateral part of the cavernous sinus. It allows wide exposure of the skull base and visualization of the dura and the cavernous sinus. The main disadvantage is its invasiveness with the risk of conductive hearing loss on the operated site, the injury of the mandibular division of the trigeminal nerve, facial asymmetry and growth retardation [9, 16, 17]. In recent years, *endoscopic approaches* have made a significant improvement in the management of JNAs. Transnasal resection has all the advantages of endoscopic surgery, such as minimal bleeding, minimal invasiveness, lower morbidity and complications rate and lower risk of recurrence in comparison to traditional approaches [18–20]. Initially indicated only in early stage angiofibromas, currently endoscopic technique is used for the management of selected cases of tumors expanding to the pterygopalatine fossa and infratemporal fossa [6, 15, 21]. Contraindications for exclusively endoscopic approach with the goal for total resection include lateral infratemporal extension, large intracranial extension, intracranial extension laterally to the cavernous sinus, encasement of the internal carotid artery (ICA), abundant supply from the ICA, significant involvement of the cavernous sinus and extension to the cheek [6, 8, 10, 22]. In endoscopic surgery, removal of the posterior wall of the maxillary sinus during surgery provides excellent exposure of the pterygopalatine fossa and medial part of the infratemporal fossa, enabling tumor extirpation. In contrast to anterior lateral growth, posterior lateral extension is more difficult to find during operation [23] (Fig. 5). This type of lateral spread, posterior to the pterygoid process, into the region of the medial and lateral pterygoid muscles, was first described by Radkowski et al. [14]. He was also the first to emphasize the importance of this extension as a factor increasing the risk of subtotal resection.

Recurrent disease is common in the management of JNAs and recurrence rates vary from 13 % to 46 % depending on tumor stage and surgical approach [20, 24, 25]. JNA is a benign lesion and many investigators agree that most recurrences can be attributed to incomplete removal [6, 8, 26, 27]. In our study recurrent disease was stated in 29.7 % (11/37) of cases. More than a half of residual tumors (7/11) were located posterior to the pterygoid process. It was the most common location of the persistent disease in the whole analyzed group and almost the only source of tumor recurrence in patients with posterior lateral extension and both types of lateral spread. The recurrence rate in patients with posterior lateral spread and both types of lateral extension was significantly higher than in the group, where only anterior lateral extension was present.

Several staging systems based on JNA extension have been proposed. In all classifications, tumor lateral extension to the pterygopalatine and infratemporal fossa reflects medium advancement of the disease—stage II and/or stage III. Radkowski et al. [14] were the first to propose a revised staging system stressing the presence of extension posterior to the pterygoid plates as a higher risk factor of persistent disease. More recently, Onerci et al. [23] suggested a modified classification system including tumor extension posterior to the pterygoid plates, which more accurately reflected the increased difficulty of complete excision (Table 3). On the basis of the significant difference in the recurrence rates, we believe that the presence of posterior lateral extension is clinically more important than the anterior lateral spread as an indicator of possible recurrence. Moreover, tumor extending posterior to the pterygoid process grows in proximity to the natural channels in the skull base, which represent the natural pathway of intracranial invasion. In our study, intracranial spread was observed in 53.3 % of patients with both anterior and posterior lateral extension, and in 4.7 % of patients with only anterior lateral spread. Our results support the classification system by Onerci et al. [23], where lateral spread posterior to the pterygoid plates represents higher stage of the disease, associated with increased risk of recurrence, than anterior lateral extension.

**Table 3:** Synopsis of staging systems for JNA

Andrews et al. 1989 [9]
(I) Tumor limited to the nasopharynx and nasal cavity. Bone destruction negligible or limited to the sphenopalatine foramen (II) Tumor invading the pterygomaxillary fossa or the maxillary, ethmoid or sphenoid sinus with bone destruction (III) A. Tumor invading the infratemporal fossa or orbital region without intracranial involvement B. Tumor invading the infratemporal fossa or orbit with intracranial extradural (parasellar) involvement (IV) A. Intracranial intradural tumor without infiltration of the cavernous sinus, pituitary fossa or optic chiasm B. Intracranial intradural tumor with infiltration of the cavernous sinus, pituitary fossa or optic chiasm
Radkowski et al. 1996 [14]
(I) A. Tumor limited to the nose and/or nasopharyngeal vault B. Extension into one or more sinuses (II) A. Minimal extension into the pterygomaxillary fossa B. Full occupation of the pterygomaxillary fossa with or without erosion of the orbital bones C. Infratemporal fossa with or without cheek or posterior to the pterygoid plates (III) A. Erosion of the skull base—minimal intracranial extension B. Erosion of the skull base—extensive intracranial extension with or without cavernous sinus invasion
Onerci et al. 2006 [23]
(I) Nose, nasopharyngeal vault, ethmoidal-sphenoidal sinuses or minimal extension to PMF (II) Maxillary sinus, full occupation of PMF, extension to the anterior cranial fossa and limited extension to the infratemporal fossa (III) Deep extension into the cancellous bone at the base of the pterygoid or the body and the greater wing of the sphenoid, significant lateral extension to the infratemporal fossa or to the pterygoid plates posteriorly or orbital region, cavernous sinus obliteration (IV) Intracranial extension between the pituitary gland and internal carotid artery, tumor localization lateral to the internal carotid artery, middle fossa extension and extensive intracranial extension

Lateral spread of JNA may be followed by two types of bony changes: pressure erosion and displacement of the pterygoid process and the posterior maxillary wall or direct involvement of cancellous bone at the base of the pterygoid process [4, 21]. The second growth pattern with further spread and involvement of the sphenoid diploë may result in the destruction of the inner table of the skull base and invasion of the middle cranial fossa.

Additionally, locally aggressive growth in this area makes complete surgical removal more difficult and increases the risk of residual/recurrent disease. Lloyd et al. [4] first indicated that the involvement of the cancellous bone at the pterygoid base is closely associated with incomplete tumor removal. He reported that 93 % of recurrences occurred in this group and multiple recurrences were associated with this type of extension. In our study the base of the pterygoid process was the second main source of tumor regrowth and the only location of the recurrent disease in patients with only anterior lateral extension. Three of 11 (27.2 %) residual tumors were observed in this location. Therefore, thorough evaluation of tumor spread at the pterygoid base is particularly important in preoperative diagnostic management. Both CT and MRI are used to determine the extent of tumor extension within the sphenoid bone. Coronal and axial plane MR images, especially with fat saturation techniques and intravenous contrast injection, are considered more accurate in depicting medullary bone involvement [21].

## Conclusions

1. Apart from the common anterior lateral extension to the pterygopalatine fossa, JNAs may present less frequent posterior lateral growth pattern along the skull base behind the pterygoid process or between the plates.
2. In tumors extending laterally the most common source of residual disease is posterior lateral extension behind/between the pterygoid plates. The recurrence rate in patients with posterior lateral extension and both types of lateral spread is significantly higher than in patients with anterior lateral extension only.
3. Both CT and MRI are valuable tools for detection of the lateral extension in the pterygopalatine and infratemporal fossa, as well as involvement of the pterygoid base. The exact relationship of posterior lateral extension, which may decrease the efficacy of minimally invasive surgery, to the eroded pterygoid process is more obvious on CT scans.
